# Identification of feature genes in intestinal epithelial cell types

**DOI:** 10.1186/s13619-024-00208-8

**Published:** 2024-11-15

**Authors:** Ruoyu Lou, Wanlu Song, Shicheng Yu, Xiaodan Wang, Yuan Liu, Ye-Guang Chen, Yalong Wang

**Affiliations:** 1 The State Key Laboratory of Membrane Biology, Tsinghua-Peking Center for Life Sciences, School of Life Sciences, Tsinghua University, Beijing, 100084 China; 2 Guangzhou National Laboratory, Guangzhou, 510005 China; 3 The MOE Basic Research and Innovation Center for the Targeted Therapeutics of Solid Tumors, Jiangxi Medical College, Nanchang University, Nanchang, 330031 China

**Keywords:** Intestinal epithelium, Stem cell, Feature genes, Cell markers

## Abstract

The intestine, is responsible for food digestion, nutrient absorption, endocrine secretion, food residue excretion, and immune defense. These function performances are based on the intricate composition of intestinal epithelial cells, encompassing differentiated mature cells, rapidly proliferative cells, and intestinal stem cells. Although the characteristics of these cell types are well-documented, in-depth exploration of their representative markers and transcription factors is critical for comprehensive cell fate trajectory analysis. Here, we unveiled the feature genes in different cell types of the human and mouse gut through single-cell RNA sequencing analysis. Further, the locations of some specific transcription factors and membrane proteins were determined by immunofluorescence staining, and their role in regulating the proliferation and differentiation of intestinal epithelial cells were explored by CRISPR/Cas9 knockout. Therefore, this study not only reports new markers for various intestinal epithelial cell types but also elucidates the involvement of relevant genes in the determination of epithelial cell fate and maintenance of stem cell homeostasis, which facilitates the tracing and functional elucidation of intestinal epithelial cells.

**Supplementary Information:**

The online version contains supplementary material available at 10.1186/s13619-024-00208-8.

## Background

The intestine is the vital organ responsible for diet digestion, nutrient absorption, hormone secretion, microbial defense and immune response (Li et al. 2021; Peterson and Artis 2014; Tremaroli and Bäckhed 2012). The maintenance of intestinal homeostasis depends on the function of epithelial cells in the mucosal layer and stromal cells distributed in the intestinal lamina propria (Goto et al. 2022; Holloway et al. 2021; McCarthy et al. 2020). The epithelial cells include mature absorptive epithelial cells, secretory goblet cells and enteroendocrine cells (EECs), intestinal stem cells (ISCs), and transit amplifying (TA) cells (Beumer et al. 2018; Schuijers et al. 2015; Serra et al. 2019; Zorn and Wells 2009). Due to the advancements of single-cell transcriptome sequencing technology, our understanding of the functionality of these cells in the intestinal epithelium and their gene expression has been greatly enhanced. Single-cell transcriptome survey of epithelial cells from different regions of murine small intestine revealed differential gene expression in enterocytes, Paneth cells (PCs), and SCs in the proximal versus distal regions, and new subsets of enteroendocrine cells and tuft cells were also identified (Grün et al. 2015; Wang et al. 2022). Through the analysis of differentially expressed genes, more novel cell markers and cell subtypes were identified in human intestine (Wang et al. 2020).

To further uncover the heterogeneity of gene expression and the related functions in mouse and human intestinal epithelial cells in great details, we analyzed the transcriptomes of 14,537 intestinal epithelial cells from the human ileum, colon and rectum (Wang et al. 2020), and 4467 epithelial cells from the mouse small intestine (Liu et al. 2023). Specifically, transcription factors (TFs), membrane proteins and cell markers of specifical cell types were selected, and their expression levels were determined in vivo. To explore their functions, we knocked down these genes in mouse organoids. Our data revealed the transcriptomic variation in various intestinal epithelial cell types of mice and human, and uncovered the variation in the expression levels of the related genes in the patients with ulcerative colitis (UC) and tumors, which would facilitate the further investigation on their functions in the inflammatory bowel disease and cancer development.

## Results

### The transcriptome profiles of specific cell markers in the mouse and human intestine

**Fig. 1:**
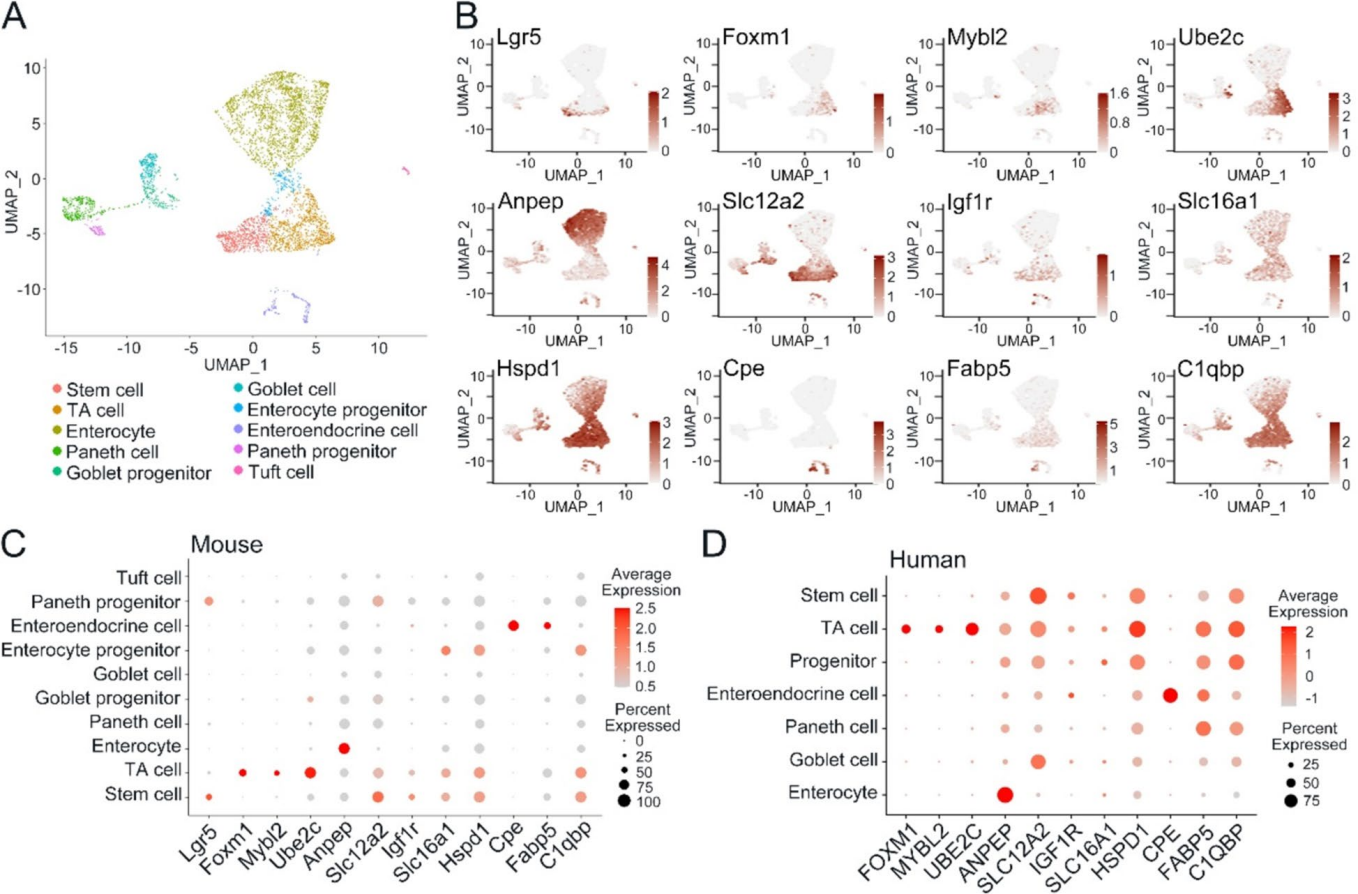
The transcriptome profiles of specific cell markers in the mouse and human intestine. **A** Cell clusters of mouse intestine based on scRNA-seq. **B** Expression (indicated by color saturation) of indicated genes in single cell of the mouse small intestine from the scRNA-seq analysis. **C** Expression patterns of the feature genes in mouse small intestinal epithelium. Each dot represents a gene, of which the color saturation indicates the average expression level (scaled by Z-core), and the size indicates the percentage of cells expressing the gene. **D** Expression patterns of the feature genes in human intestinal epithelium. Each dot represents a gene, of which the color saturation indicates the average expression level (scaled by Z-core), and the size indicates the percentage of cells expressing the gene

Based on previously published single-cell RNA sequencing (scRNA-seq) results, we categorized epithelial cells of the mouse small intestine into ten distinct cell types utilizing unsupervised graph-based clustering techniques (Fig. 1A) (Liu et al. 2023). By analyzing their transcriptome profiles of these cell groups, we screened genes exhibiting specific expression pattern within particular cell populations. These genes encode a diverse array of functional proteins, encompassing transcription regulators orchestrating the cell cycle, membrane-associated proteins pivotal in ion transport and signaling pathway activation, as well as cell markers exhibiting enriched expression in specific cell types.

During the process of cell cycle, several essential TFs play a crucial role in regulating the expression of cyclins. These regulators facilitate the mitosis progression through cell cycle checkpoints while also participate in the degradation of cyclins at specific phases of cell cycle. Consequently, these transcription factors exhibit a predominant enrichment within TA cells, while fewer levels in SCs, including *FOXM1, MYBL2* and *UBE2C* (Fig. 1B, C), which were similar in the human intestine (Fig. 1D) (Wang et al. 2020).

Among membrane-associated proteins, aminopeptidase encoded by *ANPEP* facilitates intestinal cholesterol endocytosis by binding to the enterocyte brush border membrane, which is enriched in enterocytes (Fig. 1B, C, D). Functioning as a receptor for insulin-like growth factors (IGFs), *IGF1R* is primarily expressed on the cell membrane of both SCs and TA cells. Likewise, two members of the solute carrier family genes, *SLC12A2* and *SLC16A1*, *SLC12A2* facilitates the uptake of sodium and chloride ions, while *SLC16A1* mediates the transport of monocarboxylate ions. Both genes exhibit enriched expression in SCs and TA cells, while at lower levels in absorptive enterocytes (Fig. 1B, C, D).

Apart from TFs and membrane-associated proteins, other feature genes were also identified. *HSPD1* encodes the mitochondrial molecular chaperone protein, is notably pronounced in SCs and TA cells (Fig. 1B, C, D). Similarly, mitochondrial proteins encoded by *C1QBP* participate in diverse cellular activities and exhibit notably higher in SCs and TA cells (Fig. 1B, C, D). Carboxypeptidase E (CPE), serves as an indispensable enzyme involved in hormone biosynthesis, which emerges as a gene specifically expressed in EECs (Fig. 1B, C, D). Interestingly, *FABP5*, which is instrumental in intracellular fatty acid transport and lipid metabolism, also demonstrates specific expression in EECs (Fig. 1B, C, D).

In our preliminary investigation using scRNA-seq, we identified the various cell types and expression levels of the genes of interest, and ascertained the precise expression locations and functional roles of these genes within the tissues of mouse and human intestines.

### The expression position of specific transcription factors in mouse and human intestine

**Fig. 2:**
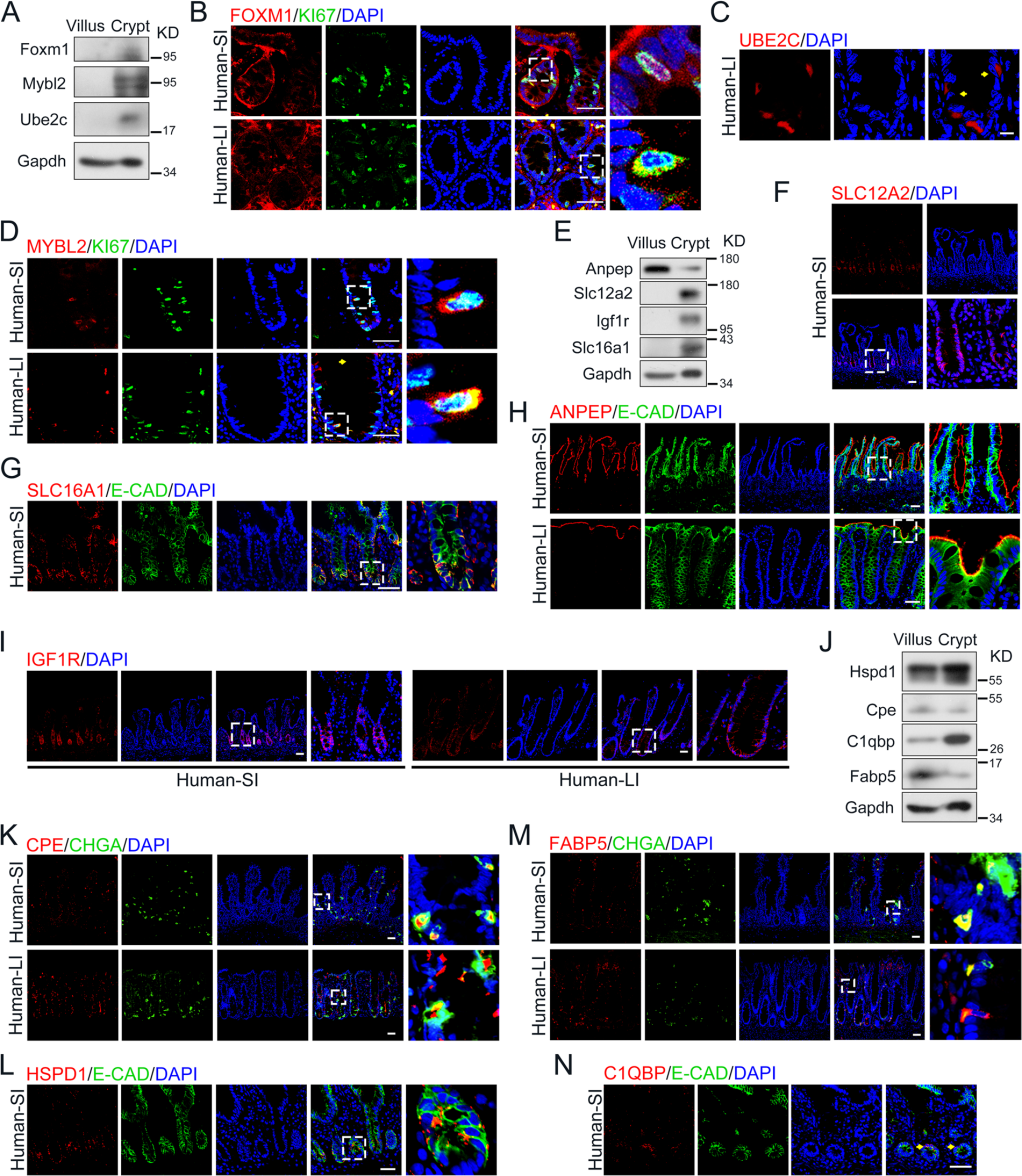
The expression of specific transcription factors, membrane-associated proteins and cell markers in human small and large intestine. **A** Immunoblot of protein FOXM1, MYBL2 and UBE2C in villus and crypt derived from mouse small intestine. **B** FOXM1 and KI67 immunostaining of small and large intestine derived from human specimens. Scale bars: 50 μm. **C** UBE2C immunostaining of large intestine derived from human specimens. Scale bars: 10 μm. **D** MYBL2 and KI67 immunostaining of small and large intestine derived from human specimens. Scale bars: 50 μm. **E** Immunoblot of protein ANPEP, SLC12A2, IGF1R and SLC16A1 in villus and crypt derived from mouse small intestine. **F** SLC12A2 immunostaining of small intestine derived from human specimens. Scale bars: 50 μm. **G** SLC16A1 and E-Cadherin immunostaining of small intestine derived from human specimens. Scale bars: 50 μm. **H** ANPEP and E-Cadherin immunostaining of small and large intestine derived from human specimens. Scale bars: 50 μm. **I** IGF1R immunostaining of small and large intestine derived from human specimens. Scale bars: 50 μm. **J** Immunoblot of protein HSPD1, CPE, FABP5 and C1QBP in villus and crypt derived from mouse small intestine. **K** CPE and Chromogranin A immunostaining of small and large intestine derived from human specimens. Scale bars: 50 μm. **L** HSPD1 and E-Cadherin immunostaining of small intestine derived from human specimens. Scale bars: 50 μm. **M** FABP5 and Chromogranin A immunostaining of small and large intestine derived from human specimens. Scale bars: 50 μm. **N** C1QBP and E-Cadherin immunostaining of small intestine derived from human specimens. Scale bars: 50 μm

Employing scRNA-seq analysis on both mouse and human ileum, our investigation unveiled distinctive expression patterns of several crucial TFs within TA cells and SCs, facilitating the intricate regulation of intestinal epithelial development, regeneration, and tumorigenesis. To precisely pinpoint their expression locations, we conducted an initial immunoblot assay, highlighting the specific expression of FOXM1, MYBL2, and UBE2C in the intestinal crypt region (Fig. 2A).

FOXM1, a transcription activator pivotal for orchestrating the expression of cell cycle genes crucial in mitosis (Fu et al. 2008; Littler et al. 2010), has also been identified as an oncogenic factor, triggering DNA damage repair, proliferation and migration of gastric cancer cells (Tan et al. 2023; Zhang et al. 2018b). Our observations revealed a pronounced expression of *FOXM1* in the nucleus of proliferative cells within both the mouse and human intestine, as evidenced by co-immunostaining with Ki67 (Fig. 2B and Fig. S1A).

As a downstream target gene of FOXM1 (Zhang et al. 2018a), ubiquitin conjugating enzyme E2 C (*UBE2C*) encodes a member of the E2 ubiquitin-conjugating enzyme family catalyzing the ubiquitination and degradation of cyclin A and B, collaborating with the E3 ligase of the anaphase-promoting complex (APC) through the spindle assembly checkpoint. To identify the expression location of UBE2C, we performed the immunostaining and immunoblot assay and confirmed the increased level of UBE2C in the crypt region of mouse and human intestine (Fig. 2C and Fig. S1B).

*MYBL2*, encoding a member belonging to the *MYB* family of TFs, is required as a pioneer factor to enable the binding of FOXM1 to G2/M gene promoters (Down et al. 2012). Suppression of MYBL2 induces a modest shift in cell-cycle distribution: a significant increase of cells accumulated in G2/M phase (Papetti and Augenlicht 2010). In both the mouse and human intestine, MYBL2 presents the predominant co-expression of KI67^+^ proliferative cells through co-immunostaining (Fig. 2D and Fig. S1C).

### The location of specific membrane proteins in mouse and human intestine

Although the functions of intestinal epithelial subtypes have been extensively studied, the identification of specific membrane proteins suitable for live cell sorting remains obscure. To address the genes responsible for encoding membrane proteins in distinct epithelial cell types, scRNA-seq analysis was performed and a repertoire of membrane-related genes were identified, predominantly including receptors crucial for signaling activation and nutrient transporters.

*SLC12A2*, a membrane cotransporter facilitating the transport and reabsorption of sodium and chloride across cellular membranes, participates in maintaining the proper ionic balance and cell volume (Payne et al. 1995; Yang et al. 2020). Analysis of scRNA-seq results indicated the increased expression of *SLC12A2* in both SCs and TA cells (Fig. 1), implying a potential impact of ion transport on the functionality of the SCs. In line with these findings, our observations consistently demonstrated a higher expression of SLC12A2 in both SCs and proliferative cells within the crypt region of mouse and human intestine tissues (Fig. 2E, F and Fig. S1D).

Similar to *SLC12A2*, *SLC16A1* expression is higher not only in the SCs, but also in absorptive enterocytes (Fig. 1). Functioning as a bidirectional proton-coupled monocarboxylate transporter, SLC16A1 catalyzes the swift transport of various monocarboxylates across the plasma membrane to the maintenance of intracellular pH (Reddy et al. 2020; Wang et al. 2021). We also confirmed that Slc16a1 exhibits predominant expression within the mouse crypt region (Fig. 2E and Fig. S1E), and the location of SLC16A1 in the basolateral membrane of human intestinal crypts (Fig. 2G).

As a receptor of F4 fimbriae, alanyl aminopeptidase (ANPEP) induced clathrin-mediated endocytosis of enterocytes in the context of the intestinal mucosal immune response (Melkebeek et al. 2012). In the intestine, *ANPEP* shows a specific higher expression in absorptive enterocytes (Fig. 1). The heightened levels of ANPEP predominantly localized to the apical membrane of enterocytes in the villus region in both mouse and human intestine tissues (Fig. 2E, H and Fig. S1F). Notably, in contrast to the human large intestine, where *ANPEP* expression is prominent, there was minimal expression of ANPEP detected in the epithelium of the mouse large intestine (Fig. S1F).

*IGF1R* encodes a high affinity receptor of insulin-like growth factor 1 (IGF1) with tyrosine kinase activity, which triggers downstream signaling pathway promoting cellular proliferation, tumor transformation, and the survival of malignant cells (Penney and Li 2018). In alignment with the notable *IGF1R* expression in SCs besides myofibroblasts based on scRNA-seq analysis, our observations underscored a heightened expression of IGF1R specifically within the crypt region (Fig. 2E, I and Fig. S1G), which may suggest the specific regulation of IGF signaling for intestinal stemness maintenance.

### The expression position of specific cell markers in mouse and human intestine

In addition to the TFs and membrane proteins mentioned above, we also screened some genes which are predominantly expressed in specific cell types through scRNA-seq analysis. The function of these genes may be involved in the regulation of proliferation and cell fate in intestinal homeostasis, thus we identified these genes as specific cell markers and determined their expression locations.

Carboxypeptidase E (CPE) is identified as a sorting receptor promoting preproprotein to generate the mature peptidase proteolytically. During hormone synthesis, *CPE* acts as a prohormone processing enzyme in endocrine cells and directs prohormones to the regulated secretory pathway. Deficiency of *CPE* suppressed the level of Neuropeptide Y (NPY) and Peptide YY (PYY), secreted by EECs (Karhausen et al. 2014). Compared with villus region, the lower level of CPE was presented in the crypt region (Fig. 2J). Consistently, CPE was mainly presented in villus region both in mouse and human intestine by co-staining with Chromogranin A (Fig. 2K and Fig. S1H).

HSPD1, as a member of the chaperonin family, prevents misfolding and promotes the refolding and proper assembly of unfolded polypeptides generated under stress conditions in the mitochondrial matrix (Levy‐Rimler et al. 2001; Viitanen et al. 1992). HSPD1 shows a lower level in the villus than in the crypts through immunoblot verification (Fig. 2J), also, the enrich expression level of HSPD1 exhibited in the SCs of crypt region in mouse and human tissues (Fig. 2L and Fig. S1I).

In prior studies, *FABP5* was highly and exclusively expressed in enteroendocrine K cells, maintaining the normal level of glucose-dependent insulinotropic polypeptide (GIP) (Sommer and Mostoslavsky 2014), which regulates diet-induced obesity in response to fat ingestion (Shibue et al. 2015). The immunoblot results showed that FABP5 is more enriched in villus compared to the crypt region in mouse intestine (Fig. 2J). Furthermore, FABP5 overlapped significantly with Chromogranin A (ChgA), which was enriched in the villus region of small intestine and top half of crypt region of large intestine in mouse and human intestines (Fig. 2M and Fig. S1J).

*C1QBP* encodes a multifunctional and multicompartmental protein, involved in inflammation process, ribosome biogenesis and protein synthesis in mitochondria (Feichtinger et al. 2017; Storz et al. 2000). *C1QBP* is broadly expressed in intestinal epithelial cells, while a larger proportion of which is enriched in SCs and progenitor cells (Fig. 1C, D), and the expression level of C1QBP was higher in the crypt region than in the villus through immunoblot verification (Fig. 2J). Furthermore, C1QBP is indeed enriched in crypt region and the bottom half of villus region of mouse and human tissues through immunostaining (Fig. 2N and Fig. S1K).

### The effect of the related genes abrogation in the mucous layer on the fate regulation of epithelial cells

To further validate the involvement of these genes in regulating the proliferation, differentiation, and apoptosis of epithelial cells, we conducted experiments involving small intestinal organoids derived from *Villin-CreERT2; Rosa26-fl-STOP-fl-Cas9*^*egfp*^ mice. These organoids were infected with adeno-associated virus (AAV) containing specific sgRNA sequences targeting the genes of interest. By employing Cas9 to target the corresponding sgRNA sequences, we effectively disrupted the normal transcriptional processes within the epithelial cells.

**Fig. 3:**
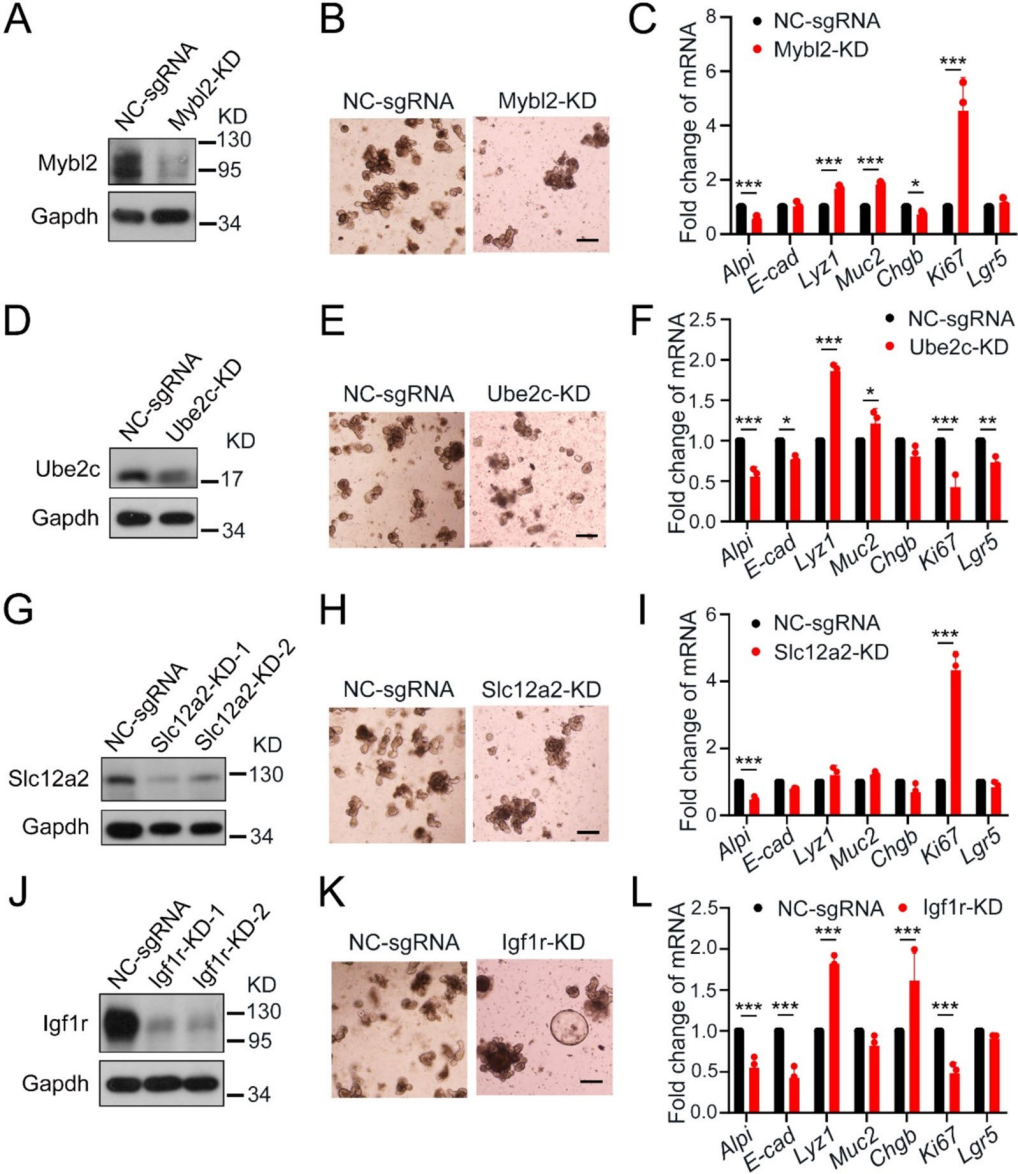
Functional validation of genes specifically expressed in stem cells and proliferative cells in the small intestine epithelium. **A** Immunoblot of the level of Mybl2 in small intestinal organoids after AAV infection. **B** Representative images of small intestinal organoids with *Mybl2*-knockdown at day 4 after AAV infection. Scale bars: 200 μm. **C** qRT-PCR of epithelial marker genes in small intestinal organoids with *Mybl2*-knockdown at day 4 after AAV infection. **D** Immunoblot of the level of Ube2c in small intestinal organoids after AAV infection. **E** Representative images of small intestinal organoids with *Ube2c*-knockdown at day 4 after AAV infection. Scale bar: 200 μm. **F** qRT-PCR of epithelial marker genes in small intestinal organoids with *Ube2c*-knockdown at day 4 after AAV infection. **G** Immunoblot of the level of Slc12a2 in small intestinal organoids after AAV infection. **H** Representative images of small intestinal organoids with *Slc12a2*-knockdown at day 4 after AAV infection. Scale bar: 200 μm. **I** qRT-PCR of epithelial marker genes in small intestinal organoids with *Slc12a2*-knockdown at day 4 after AAV infection. **J** Immunoblot of the level of Igf1r in small intestinal organoids after AAV infection. **K** Representative images of small intestinal organoids with *Igf1r*-knockdown at day 4 after AAV infection. Scale bar: 200 μm. **L** qRT-PCR of epithelial marker genes in small intestinal organoids with *Igf1r*-knockdown at day 4 after AAV infection. *p < 0.05, ** *p* < 0.01, ***p < 0.001. Data are displayed as the mean ± SD by two-way ANOVA in (**C**), (**F**), (**I)** and (**L**)

As an upstream regulator facilitating the binding of the FOXM1 transcription factor to its promoter region, Mybl2 plays a crucial role in the regulation of epithelial cell proliferation. Upon knockdown of *Mybl2*, a marked depletion of its protein levels was observed in small intestinal organoids derived from mice (Fig. 3A), so as a significant decrease in the number of budding organoids and an increase in apoptotic cells (Fig. 3B). Furthermore, *Mybl2* reduction significantly promoted the proliferation and differentiation of secretory cells (Fig. 3C). Meanwhile, diminished expression of *Ube2c* led to a notable reduction in organoid size and budding number (Fig. 3D, E). Moreover, the lower levels of *Ube2c* significantly inhibited proliferation and stemness while promoting Paneth cell differentiation within the epithelium (Fig. 3F).

Loss of *Slc12a2*, functioning as a membrane-associated transporter in SCs and TA cells, significantly promoted proliferation and inhibited the differentiation of absorptive epithelial cells (Fig. 3G, H, I). Furthermore, as a receptor displaying high-affinity binding to the Igf1 ligand, decreased expression of *Igf1r* resulted in the inactivation of the IGF signaling pathway (Fig. 3J), alongside an increase in the size of spheroids (Fig. 3K). Notably, the inactivation of Igf1r significantly suppressed the proliferation and differentiation of absorptive enterocytes, while leaving the differentiation of secretory cells unaffected (Fig. 3L).

### The expression levels of the feature genes in human colitis and colorectal cancer

After delineating the expression patterns of these genes in mouse and human intestine tissues, our research also focused on whether these genes are implicated in the onset of inflammation and the progression of tumors. To achieve this, we are conducting an analysis of the transcriptome variations of the corresponding genes in specimens obtained from patients diagnosed with UC and colorectal cancer (CRC).

Previous research has highlighted an upregulated expression of *IGF1R* in primary gastrointestinal sarcomas (Lasota et al. 2013). Additionally, knockdown of *IGF1R* promotes apoptosis in CRC cells through the activation of the mitochondrial pathway by triggering the expression of MDA5 and RIG-I in the intestinal epithelium (Wang et al. 2019). Our investigation further reveals a significant reduction in the expression of *IGF1R* in patients diagnosed with UC (Fig. S2A). Intriguingly, despite this reduction in expression, the activation level of the IGF signaling pathway displays variability across primary CRC (Fig. S2B).

In both UC and CRC patients, the absence of *SLC16A1* disrupts the transport of bacterial products via H^+^-coupled short-chain fatty acid transport mechanisms (Sivaprakasam et al. 2017). Furthermore, our observations indicate a notable suppression in the levels of *SLC16A1* in both UC patients and primary CRC (Fig. S2A, B).

Additionally, loss of ANPEP has been shown to mitigate the clinical manifestations of colitis by dampening the activity of both activated and regulatory T cells (Bank et al. 2006). Consistently, the lower expression levels of *ANPEP* observed in primary tumor tissues of CRC and UC patients further substantiate this phenomenon (Fig. S2A, B).

Abundant intraepithelial expression of *HSPD1* during the early stages of CRC has been found to inhibit apoptosis by facilitating the binding of pro-caspase 3, thereby supporting cancer cell survival (C. Campanella and A.M. Czarnecka 2008; Rappa et al. 2016). Moreover, aside from the heightened expression levels observed in tumors, we have also identified a significant enrichment of *HSPD1* in inflammatory samples (Fig. S2C, D).

The novel isoform of *FOXM1* has been shown to directly interact with ROCK2, thereby activating Rho/ROCKs signaling, which further promotes actin polymerization and impedes E-cadherin expression, ultimately culminating in epithelial-mesenchymal transition (EMT) and metastasis in CRC (Zhang et al. 2016). In line with findings from tumor samples, *FOXM1* expression was found to be enriched in colitis samples as well (Fig. S2C, D), suggesting its pivotal role in driving malignant proliferation of tumor cells.

Similarly, upregulation of *UBE2C* has been associated with the amplification of copy number in chromosome 20q and activation of MAPK1 signaling, thereby promoting cell growth and invasive abilities of gastric cancer cells (Nicolau-Neto et al. 2018). Similarly, a high enrichment of *UBE2C* in primary tumor cells was observed (Fig. S2D). Moreover, the elevated expression of *UBE2C* in inflammatory samples suggests that damaged tissues undergo regenerative repair by driving hyperproliferation (Fig. S2C).

To evaluate the impact of signature gene expression on patient survival, various tumor types were further analyzed, including colon adenocarcinoma, stomach adenocarcinoma, and lung adenocarcinoma (Gyorffy 2024a, b; Tang et al. 2019, 2017). High expression of transcription factors *FOXM1*, *MYBL2*, and *UBE2C* (Fig. S3A, C, E) was associated with better survival in colon cancer patients, yet showed the opposite effect in stomach and lung cancers (Fig. S3B, D, F), aligning with our findings. Lower *CPE* expression correlated specifically with better survival in stomach adenocarcinoma, while showed no impact on the survival in both colon and lung cancers (Fig. S3G, H).

Although *HSPD1* was upregulated in all three adenocarcinomas, its association with reduced survival in colon cancer and better survival in lung cancer (Fig. S3I, J). Similarly, *ANPEP* showed higher progression in stomach cancer and reduced survival in stomach cancer, and lower expression levels in colon and lung tumors with reduced survival in colon patients (Fig. S3K, L). Overall, these cell markers display distinct expression profiles and effects across tumor types, variably influencing progression and patient survival.

## Discussion

As an organ capable of food digestion, nutrient absorption, immune defense and hormone secretion, intestine growth and development in homeostasis, damage repair and tumor formation under pathological conditions have been extensively studied. With the continuous progress of single-cell transcriptome sequencing technology, we have gained a deeper insight of the gene expression pattern variation between different intestinal segments, different cell types, and in pathological tissues (Liu et al. 2023; Wang et al. 2020), which promotes us to further explore the regulation of cell differentiation fate during intestinal development and the essential genes in intestinal inflammation and tumorigenesis.

However, the function of some genes specifically expressed in certain cell types in the cell fate regulation of epithelial cells, inflammation and tumorigenesis is not well understood. In this study, we screened and validated some genes that are enriched expressed in specific cell types, including transcription factors regulating cell cycle, membrane-associated proteins involved in nutrient transport and signaling activation, and cell markers related with functions of certain cell population. In addition, we verified the function of the related genes in epithelial cell fate regulation by gene knockdown in small intestinal organoids, and further explored the possible role of these genes in inflammation and tumorigenesis by using pathological samples of patients.

ScRNA-seq analysis revealed that FABP5 is enriched in EECs of the mouse intestinal epithelium, whereas in humans, it is more broadly expressed across EECs. This expression difference was further confirmed by immunofluorescence staining. C1QBP is broadly expressed in various epithelial cell types at the mRNA level in both human and mouse intestine, yet in human intestinal epithelium, protein staining shows it is primarily enriched in the stem cell region. SLC12A2 is highly expressed in human stem cells and secretory cells, but in mice, it is mainly expressed in stem and TA cells. Immunostaining verified high SLC12A2 expression in stem and proliferative cells for both species. In both human and mouse intestinal epithelium, HSPD1 shows low expression specificity, being abundant in the stem cell zone and widely distributed across mature cells. These specificity variations should be further studied to validate the representativeness of these markers.

Our data confirmed that inhibition of MYBL2 would promote a large number of proliferative cells to be stagnated in the G2/M phase, which is consistent with some earlier research, but as a transcription factor driving cells through the cell cycle checkpoint, UBE2C suppression induces the proliferation blockade and variation in the differentiation direction of epithelial cells. However, our data only verified the effect of the related genes deletion on the proliferation and differentiation of small intestinal epithelial cells at the transcriptional level, and did not reveal the effect of gene knock-down on the downstream regulated protein and some signaling pathways, further affecting the decision of cell differentiation fate. Also, inhibition of the IGF signaling pathway in organoids also revealed a transient damage repair process in organoids, which is similar to the rapid expansion during the fetal-like state (Serra et al. 2019). In addition, bioinformatics analysis revealed differences in the expression of the related genes in patient samples, but the specific regulatory mechanisms of these genes during inflammation and tumorigenesis remain unclear. Exploring the expression level variation of these genes and the alterations in the related signaling pathways in mouse models would help to better understand the role of these genes in the development of human diseases.

## Conclusions

By analyzing scRNA-seq data and validating through immunofluorescence staining, we identified the expression levels of feature genes (*Hspd1, Cpe, C1qbp, Fabp5*), key transcription factors (*Foxm1, Ube2c, Myble2*) and membrane proteins (*Anpep, Slc12a2, Igf1r, Slc16a1*) across different intestinal epithelial cell types. Their functions were further confirmed through gene knockdown experiments in intestinal organoids, highlighting their roles in intestinal inflammation and tumor development.

## Materials and methods

### Human sample analysis

TCGA-COAD gene expression data and clinical information were obtained using GDCRNATools (access date: Sep 27, 2023), which facilitated the organization and integrative analysis of RNA expression data in the Genomic Data Commons (GDC) Data Portal (Liu et al. 2023; Nicolau-Neto et al. 2018). Microarray data were derived from Gene Expression Omnibus (GEO) dataset [GSE38713 (Planell et al. 2013) and GSE53306 (Zhao et al. 2015)]. The datasets were directly accessed through the R (v4.2.1) package GEOquery to retrieve normalized expression data. Then, the R (v4.2.1) package ggpubr (https://cran.r-project.org/web/packages/ggpubr/index.html) was used to generate box plots and calculate respective P value, and Wilcoxon rank sum tests were used to compare two conditions.

### Mice

*Villin-CreERT2* mice were a gift from Dr. Sylvie Robine (Institute Curie-CNRS, Paris), and *Rosa26-loxp-stop-loxp-Cas9-EGFP* mice from Dr. Jianwei Wang (Tsinghua University). All mice were performed as previously described (Wang and Liu 2023). All animal studies were performed in accordance with the guidelines and under the approval of the Institutional Animal Care and Use Committee of Tsinghua University (YGC-19).

### Immunofluorescence staining

Intestinal tissues were fixed with 4% formaldehyde solution for overnight and dehydrated with gradient concentration ethanol. Then, tissues were embedded in paraffin with paraffin embedding machine (Arcadia H + C; Leica), and sections were prepared with paraffin slicing machine (RM2255; Leica). For frozen sections, intestine tissues were fixed with 4% formaldehyde solution for 2 h at 4 °C, followed by dehydrating in 30% sucrose solution at 4 °C overnight. Next, the tissues were embedded in optimal cutting temperature compound (Sakura) and stored at -80 °C. Sections were prepared with freezing slicing machine (CM1950; Leica). For staining, the sections were permeabilized with PBST solution (3% bovine serum albumin and 0.1% Triton X-100 in PBS) and then incubated overnight with the primary antibody at 4 °C. The fluorescein-labeled secondary antibodies (1:800; the Jackson Laboratory) for immunofluorescence or secondary horseradish peroxidase conjugated goat anti-rabbit antibody (ZSGB-BIO) for immunohistochemistry were added for 2 h at room temperature. Confocal laser scanning (FV3000; Olympus) or 3,30-diaminobenzidine chromogenic fluid (ZSGBBIO) was used to detect the staining signals.

### Antibodies

Mouse anti-Ki67 (1:200; 9449, CST), mouse anti-E-cadherin (1:1000; 610,182, BD Biosciences), mouse anti-chromogranin A (1:200; sc-393941, Santa Cruz), rabbit anti-Foxm1 (1:100; ab207298, Abcam), rabbit anti-Ube2c (1:50; 12,134–2-AP, Proteintech), rabbit anti-Mybl2 (1:100; ab76009, Abcam), rabbit anti-Anpep (1:50; 14,553–1-AP, Proteintech), rabbit anti-Slc12a2 (1:50; 13,884–1-AP, Proteintech), rabbit anti-Slc16a1 (1:50; 20,139–1-AP, Proteintech), rabbit anti-Igf1r (1:100; ab182408, Abcam), rabbit anti-Cpe (1:50; 13,710–1-AP, Proteintech), rabbit anti-Hspd1 (1:50; 15,282–1-AP, Proteintech), rabbit anti-C1qbp (1:50; 24,474–1-AP, Proteintech), rabbit anti-Fabp5 (1:100; ab255276, Abcam).

### qRT-PCR analysis

The cultured organoids were precipitated, and RNA was purified using the RNeasy Kit (mf167-01, Mei5 Biotech) and converted into cDNA using the NovoScript One-Step RT-PCR kit (Novoprotein). Real-time PCR reactions were performed in triplicates on a LightCycler 480 (Roche).

### Virus production and organoid infection

*Villin-CreERT2;Rosa26loxp-stop-loxp-Cas9-EGFP* organoids were pretreated with 5 μM 4-OHT to induce Cas9 expression. Recombinant adeno-associated virus (AAV) were produced as previously described (Liu et al. 2023). Before virus infection, organoids were cultured with the expansion medium with 10 mM nicotinamide for 3 days. Then, the organoids were digested with TrypLE (Gibco, 12,604,021) and re-suspended with the expansion medium with 10 mg/mL polybrene (Macgene, MC032) containing virus. We added 250 mL of expansion medium plus polybrene containing cells and virus on the pre-solidified Matrigel and incubated overnight at 37 ℃. And then, we removed the medium and washed the virus with warm PBS. Then, we overlaid 15 μL Matrigel and cultured the organoids with expansion medium.

### Single-cell RNA-seq analysis

The single-cell RNA-seq of mouse intestine has been reported (Liu et al. 2023). Briefly, Raw reads were aligned to the GRCm38/mm10 mouse genome, and Cell Ranger (v3.1.0) was used to estimate unique molecular identifiers. Raw aligned features were loaded and processed using the Seurat package (v4.0.2) in R version 4.0.5. Low-quality cells were filtered if they expressed no more than 200 genes or with more than 20% of mitochondrial genes. Then, the unsupervised clustering and gene expression were analyzed.

## Supplementary Information

Supplementary Material 1. Supplementary Table. Primers used for qRT-PCR analysis. Fig S1. The expression of specific transcription factors, membrane proteins and cell markers in mouse small and large intestine. Fig S2. The expression levels of the related membrane proteins and cell markers in human colitis and primary tumor samples from colorectal cancer. Fig S3. The expression levels of relevant cell markers in three cancer types and their association with patient survival across various tumors.

## Data Availability

All data needed to evaluate the conclusions in the paper are present in the paper and/or the Supplementary Materials.

## Abbreviations

CRISPR: Clustered Regularly Interspaced Short Palindromic Repeats
FOXM1: Forkhead box M1
MYBL2: MYB proto-oncogene like 2
UBE2C: Ubiquitin conjugating enzyme E2 C
ANPEP: Alanyl aminopeptidase
IGF1R: Insulin like growth factor 1 receptor
SLC12A2: Solute carrier family 12 member 2
SLC16A1: Solute carrier family 16 member 1
HSPD1: Heat shock protein family D (Hsp60) member 1
C1QBP: Complement C1q binding protein
FABP5: Fatty acid binding protein 5
Ki67: Marker of proliferation Ki-67
MDA5(IFIH1): Interferon induced with helicase C domain 1
RIG-I: RNA sensor RIG-I
Rho: Rhodopsin
ROCKs: Rho associated coiled-coil containing protein kinase
MAPK1: Mitogen-activated protein kinase 1
NC: Negative Control
KD: Knock down
