# Supplementary material for: Identification of feature genes in intestinal epithelial cell types
Source: Cell Regen. 2024 Nov 15;13:24. doi: 10.1186/s13619-024-00208-8 (PMC11564585; doi:10.1186/s13619-024-00208-8)
Supplement: Supplementary file 1 — Supplementary Material 1. Supplementary Table. Primers used for qRT-PCR analysis. Fig S1. The expression of specific transcription factors, membrane proteins and cell markers in mouse small and large intestine. Fig S2. The expression levels of the related membrane proteins and cell markers in human colitis and primary tumor samples from colorectal cancer. Fig S3. The expression levels of relevant cell markers in three cancer types and their association with patient survival across various tumors. [file 13619_2024_208_MOESM1_ESM.docx]

**Supplementary information**

**Identification of feature genes in intestinal epithelial cell types**

Ruoyu Lou^1^, Wanlu Song^1^, Shicheng Yu^2^, Xiaodan Wang^1^, Yuan Liu^1^, Ye-Guang Chen^1, 2, 3 #^ and Yalong Wang^2 #^

1 The State Key Laboratory of Membrane Biology, Tsinghua-Peking Center for Life Sciences, School of Life Sciences, Tsinghua University, Beijing, 100084, China

2 Guangzhou National Laboratory, Guangzhou 510005, China

3 The MOE Basic Research and Innovation Center for the Targeted Therapeutics of Solid Tumors, Jiangxi Medical College, Nanchang University, Nanchang 330031, China

# Corresponding author: Ye-Guang Chen ([ygchen@tsinghua.edu.cn](mailto:ygchen@tsinghua.edu.cn)) and Yalong Wang ([wangyl16@tsinghua.org.cn](mailto:wangyl16@tsinghua.org.cn))

**Supplemental Table**

**Supplemental Figures**

**Supplementary Table**

Primers used for qRT-PCR analysis

| **Gene**  **name** | **Forward sequence (5'-3')** | **Reverse sequence (5'-3')** |
| --- | --- | --- |
| *Alpi* | GGTCAAGGCCAACTACAAGA | CACGGTACATCACTGAGAAGAC |
| *Muc2* | CTACCACCATTACCACCACTAC | GTCTCTCGATCACCACCATTT |
| *Lyz* | CAGCCCATTCTGTCTCTTTCT | TCTGCTGAAGTCCTGTTACTTG |
| *Chgb* | GTGAAGAAACCGAGGAAGAGAG | TGGTGTCAAGAGCAGAATGG |
| *E-Cad* | CTGCTGCTCCTACTGTTTCTAC | TCTTCTTCTCCACCTCCTTCT |
| *Ki67* | AGGCTCCGTACTTTCCAATTC | CGTCTTAAGGTAGGACTTGCAG |
| *Lgr5* | CGTAGGCAACCCTTCTCTTATC | GCACCATTCAAAGTCAGTGTTC |
| *GAPDH* | AACAGCAACTCCCACTCTTC | CCTGTTGCTGTAGCCGTATT |


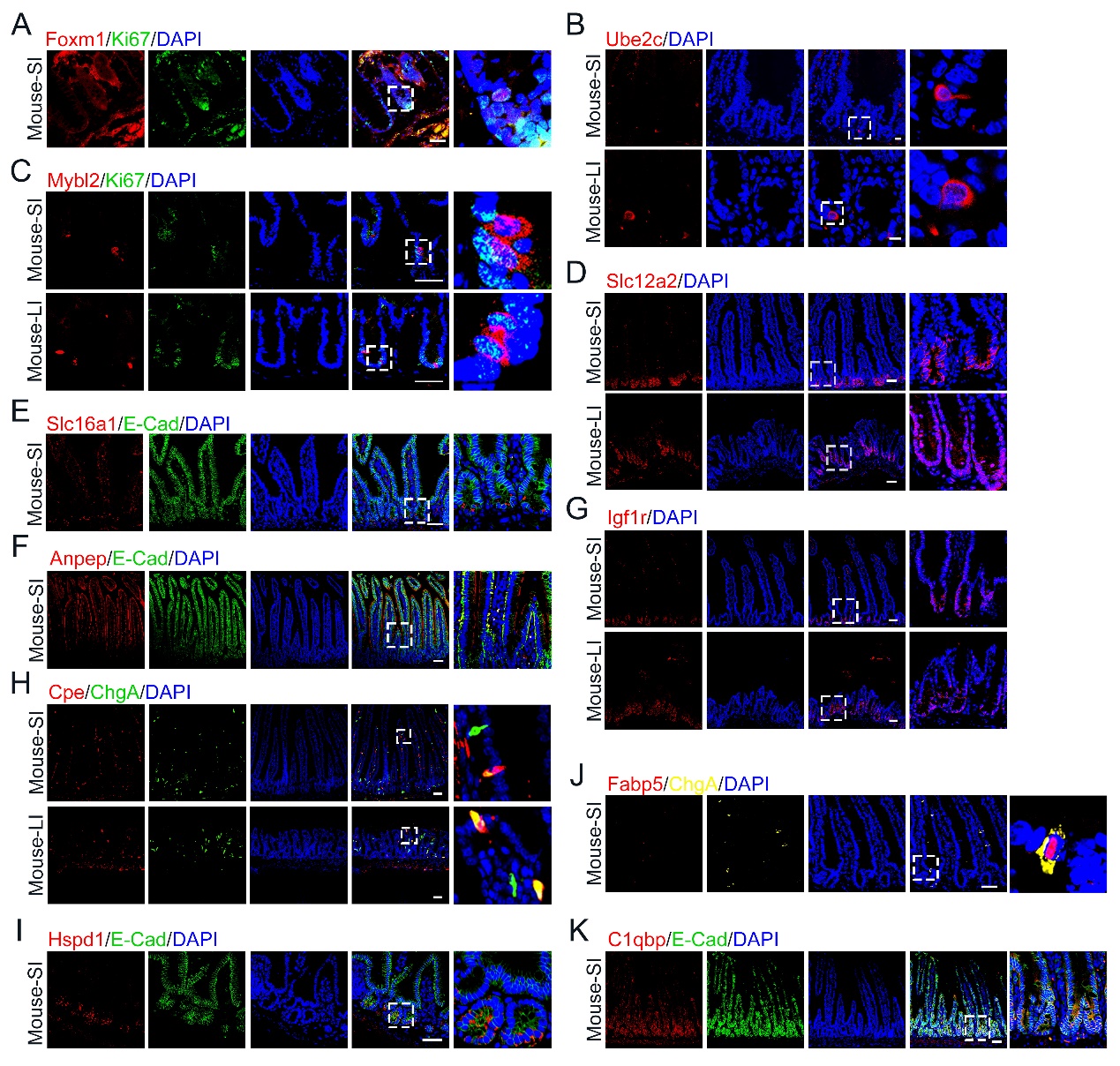


**Fig S1. The expression of specific transcription factors, membrane proteins and cell markers in mouse small and large intestine.**

1. Foxm1 and Ki67 immunostaining of small intestine derived from mouse. Scale bars: 50 μm.
2. Ube2c immunostaining of small and large intestine derived from mouse. Scale bars: 10 μm.
3. Mybl2 and Ki67 immunostaining of small and large intestine derived from mouse. Scale bars: 50 μm.
4. Slc12a2 immunostaining of small and large intestine derived from mouse. Scale bars: 50 μm.
5. Slc16a1 and E-Cadherin immunostaining of small intestine derived from mouse. Scale bars: 50 μm.
6. Anpep and E-Cadherin immunostaining of small intestine derived from mouse. Scale bars: 50 μm.
7. Igf1r immunostaining of small and large intestine derived from mouse. Scale bars: 50 μm.
8. Cpe and Chromogranin A immunostaining of small and large intestine derived from mouse. Scale bars: 50 μm.
9. Hspd1 and E-Cadherin immunostaining of small intestine derived from mouse. Scale bars: 50 μm.
10. Fabp5 and Chromogranin A immunostaining of small intestine derived from mouse. Scale bars: 50 μm.
11. C1qbp and E-Cadherin immunostaining of small intestine derived from mouse. Scale bars: 50 μm.


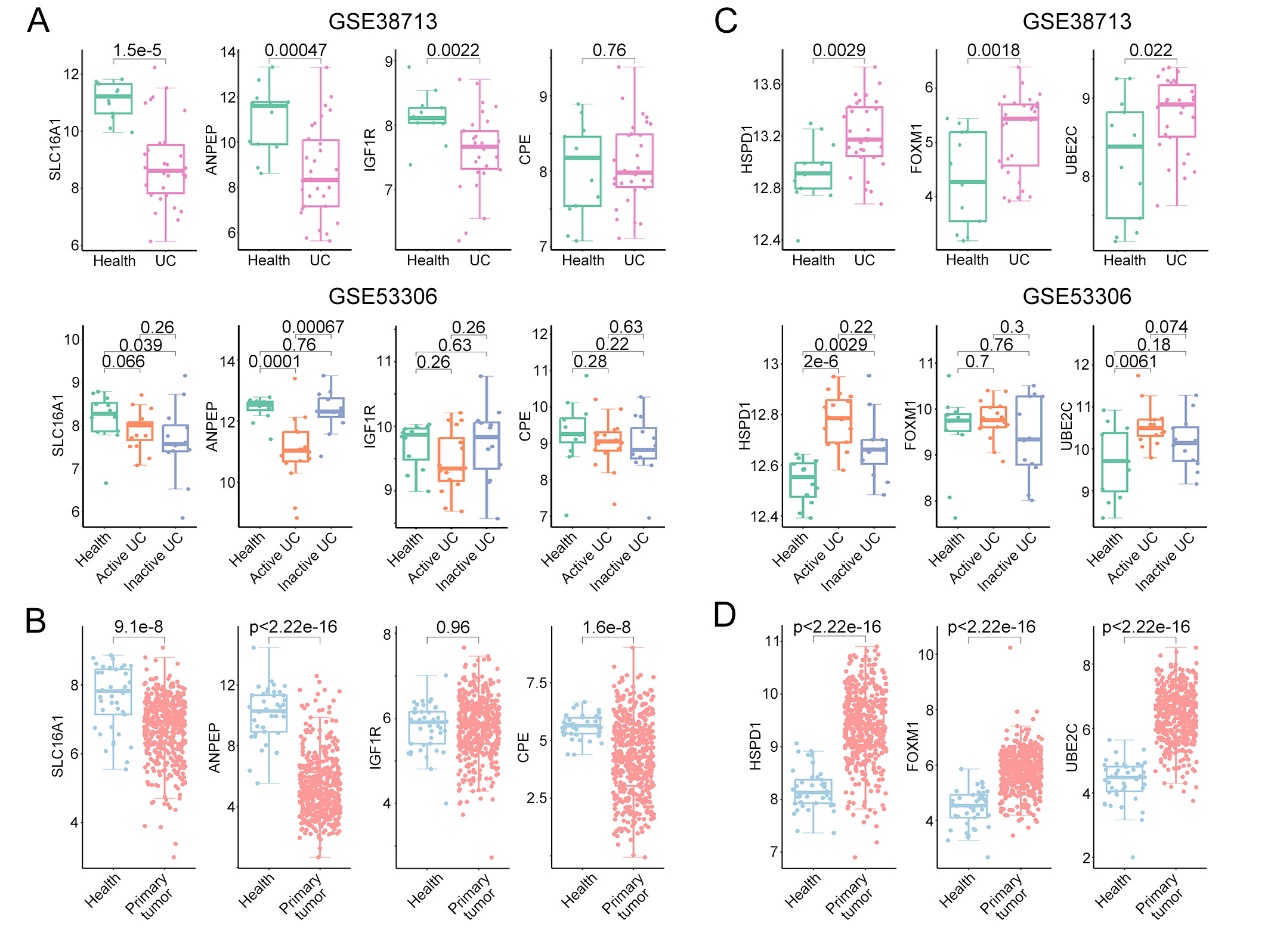


**Fig S2. The expression levels of the related membrane proteins and cell markers in human colitis and primary tumor samples from colorectal cancer.**

1. Box plots of decreased mRNA levels of *SLC12A1*, *ANPEP*, *IGF1R* and *CPE* in colitis specimens compared to healthy group (using datasets GSE38713 and GSE53306).
2. Box plots of mRNA levels of *SLC12A1*, *ANPEP*, *IGF1R* and *CPE* in healthy and tumor specimens.
3. Box plots of increased mRNA levels of *HSPD1*, *FOXM1* and *UBE2C* in colitis specimens compared to healthy group (using datasets GSE38713 and GSE53306).
4. Box plots of mRNA levels of *HSPD1*, *FOXM1* and *UBE2C* in healthy and tumor specimens.

In the box plots, the middle line depicts the median and the whiskers in the min-to-max range. The P values were calculated using T-test method using stat_compare_means function in ggpubr (0.6.0) package.


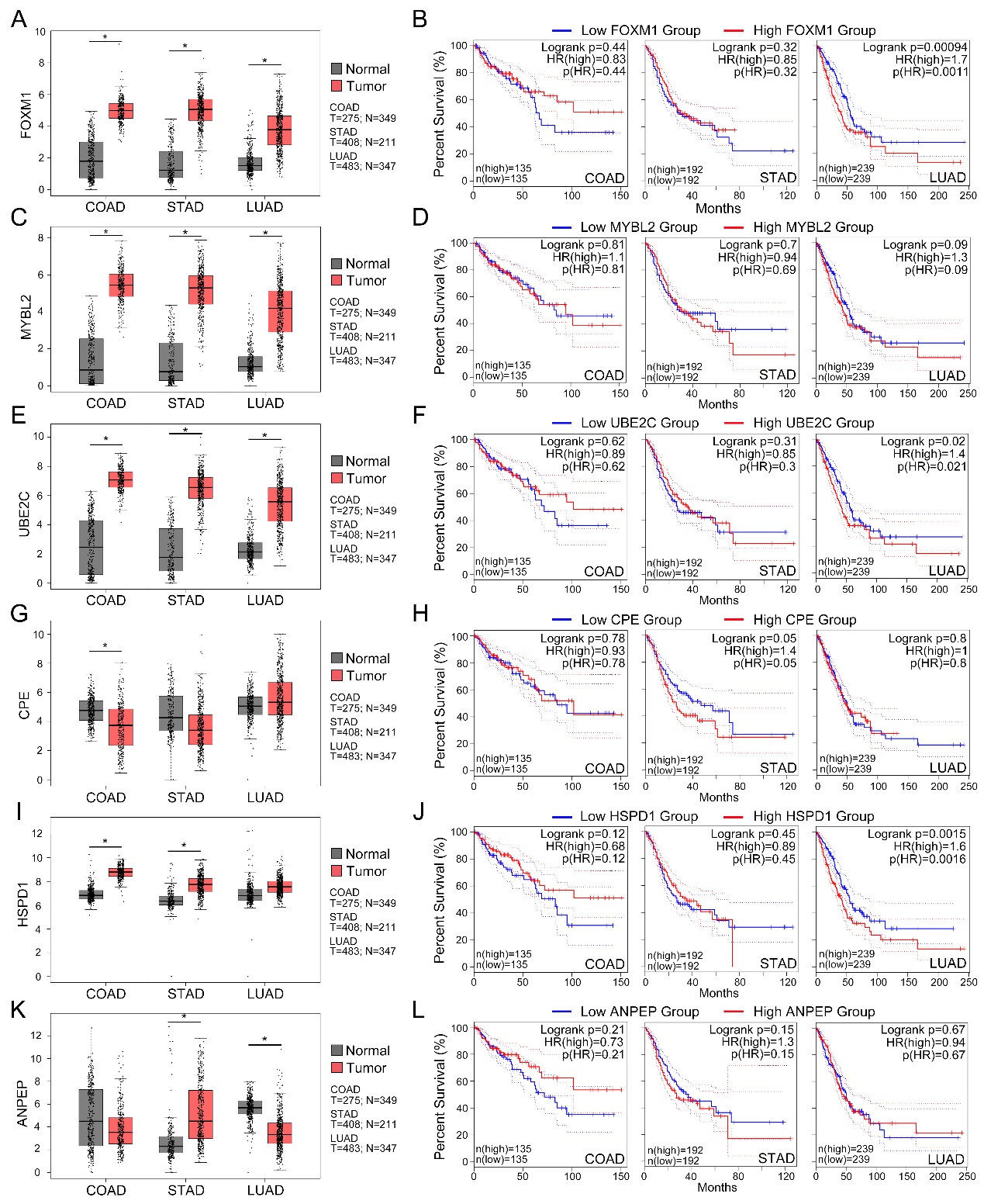


**Fig S3. The expression levels of relevant cell markers in three cancer types and their association with patient survival across various tumors.**

(A, C, E, G, I, K) Box plots showing related mRNA levels of *FOXM1* (A), *MYBL2* (C), *UBE2C* (E), *CPE* (G), *HSPD1* (I), *ANPEP* (K) across three cancer types compared to their respective normal groups (using TCGA normal and GTEx datasets).

(B, D, F, H, J, L) Kaplan-Meier analysis of the impact of expression levels of *FOXM1* (B), *MYBL2* (D), *UBE2C* (F), *CPE* (H), *HSPD1* (J), *ANPEP* (L) on patient survival across three tumor types.

COAD: colon adenocarcinoma; STAD: stomach adenocarcinoma; LUAD: lung adenocarcinoma. In the box plots, the middle line depicts the median and the whiskers in the min-to-max range.
